# A de novo heterozygous variant in the *SON* gene is associated with Zhu‐Tokita‐Takenouchi‐Kim syndrome

**DOI:** 10.1002/mgg3.1496

**Published:** 2020-09-14

**Authors:** Lianlian Yang, Fan Yang

**Affiliations:** ^1^ Department of Child Health West China Second University Hospital Sichuan University Chengdu China; ^2^ Key Laboratory of Birth Defects and Related Diseases of Women and Children Ministry of Education Sichuan University Chengdu China

## Abstract

**Background:**

Zhu‐Tokita‐Takenouchi‐Kim (ZTTK, OMIM# 617140) syndrome is a rare, autosomal dominant genetic disorder caused by heterozygous variants in the *SON* gene (OMIM#182465, GenBank#NC_000021.9). There are only 33 cases and 26 causative *SON* variants reported to date since the first report in 2015. Here, we report a new case of ZTTK syndrome and a de novo disease‐causing *SON* variant.

**Methods:**

We conducted whole‐exome sequencing (WES) to obtain genetic data of the patient. The clinical and genetic data of the patient were analyzed.

**Results:**

The clinical features of our patient were strikingly similar to previously reported cases. Notably, our patient had unique presentations, including a bridged palmar crease in the left hand and growth hormone deficiency. The c.5297del de novo variant in *SON* causes an amino change (p.Ser1766Leufs*7).

**Conclusion:**

Our report expands the mutant spectrum of the *SON* gene and refines the genotype‐phenotype map of ZTTK syndrome. Our findings also highlighted the importance of WES for early diagnosis of ZTTK syndrome, which may improve diagnostic procedures for affected individuals.

## Graphical abstract

There are only 33 cases and 26 causative variants in SON of ZTTK syndrome have been reported to date worldwide, and we reported 1 additional case of ZTTK syndrome and revealed 1 de novo disease‐causing variants in SON. Our patient had unique presentations, including a bridged palmar crease in the left hand and growth hormone deficiency. Our report expands the mutant spectrum of SON gene and refine the genotype‐phenotype map of ZTTK syndrome.

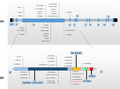

## 1 INTRODUCTION

**FIGURE 1:**
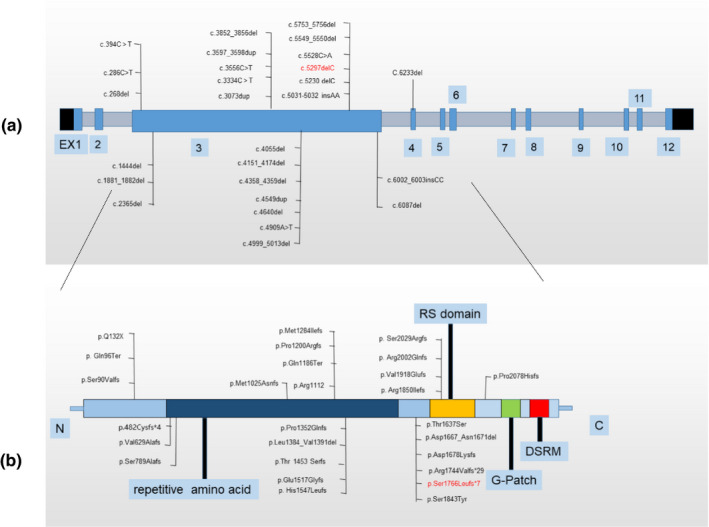
Variants of the ZTTK syndrome reported in literatures to date. Red words are the variant of present report; DSRM: double‐stranded RNA‐binding motif; SON, GenBank: NC_000021.9 (a) Nucleotide changes caused by variants of SON gene; (b) Amino changes caused by variants of SON gene.

Zhu‐Tokita‐Takenouchi‐Kim (ZTTK, OMIM#617140) syndrome is an autosomal dominant hereditary disease caused by heterozygous variants in the *SON* gene (OMIM#182465, GenBank#NC_000021.9) located on chromosome 21q22.11. There are only a few cases reported to date (Zhu et al., 2015; Tokita et al., 2016; Takenouchi, Miura, Uehara, Mizuno, & Kosaki, 2016; Kim, Baddoo, et al., 2016; Kim, Shinde, et al. 2016; Quintana et al., 2020; Tan et al., 2020; Yang, Xu, Yu, Huang, & Yang, 2019). It was first recognized in 2015 by Zhu et al, who described a 5‐year‐old girl with developmental delay, epilepsy, mild malformation, megalencephaly, white matter dysplasia, intestinal atresia, and ventricular septal defect. A new heterozygous frameshift variant, c.5753_5756del with 4 bp deletion, which results in a premature termination codon, was detected in *SON* (Zhu et al., 2015). In 2016, Takenouchi et al. reported the same frameshift variant at the same locus in *SON* in a boy with similar manifestations. Tokita et al. described seven unrelated individuals with de novo variants in *SON* and revealed that these variants are associated with a severe multisystem disorder. In 2016 (Kim, Baddoo, et al., 2016; Kim, Shinde, et al. 2016) compared the phenotypic characteristics of 20 individuals with pathogenic variants and identified de novo loss‐of‐function mutations in *SON* as a cause of complex neurodevelopmental disorder. Kim et al. also revealed that SON haploinsufficiency results in defective RNA splicing of multiple genes critical for neural development. There are 33 cases of ZTTK syndrome reported to date worldwide (except the present case), and 26 causative *SON* variants have been identified so far (Figure 1).

Here, we report an additional ZTTK syndrome case and a de novo heterozygous variant in *SON*, c.5297del (p.Ser1766Leufs*7), detected using diagnostic whole‐exome sequencing (WES; Figure 3). The clinical manifestations of our patient were similar to those in previously reported cases. In this study, we described the clinical features of our patient and report a de novo variant underpinning this condition.

## 2 CLINICAL DESCRIPTION

Our patient was an 11 years and 4 months old girl born at full‐term through spontaneous labor, with oligohydramnios and placenta senility. The propositus was small‐for‐gestational‐age (SGA) infant with a birth weight of 2200 g and a body length at birth of 47 cm. Feeding difficulties occurred shortly after birth. Her psychomotor and language development were significantly delayed. The patient began walking and saying “mummy” and “daddy” at about 2 years of age. She still has severe dysarthria and was 128.2 cm (<P_3,_ <−3SD) in height. The target height of the patient is 152.76 ± 3 cm (The patient's father and mother are 165 and 158 cm in height, respectively).

### 2.1 Physical examination

**FIGURE 2:**
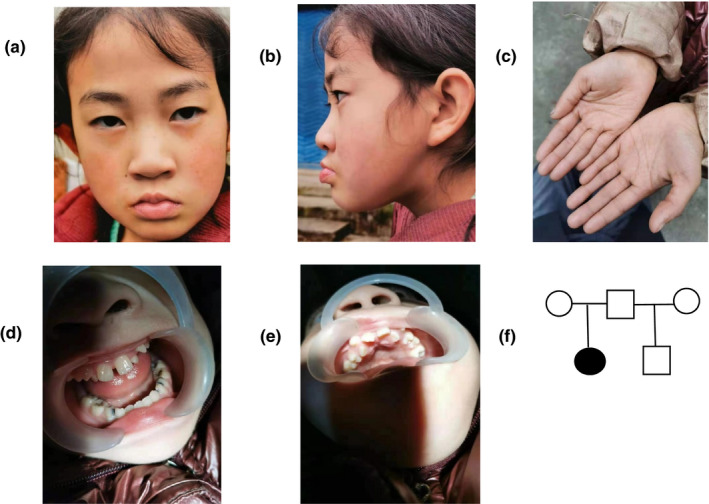
Clinical features of patient. (a) (b) (d) (e) High‐arched palate, widely spaced teeth with malocclusion. (c) Bridged palmar crease of left hand. (f) Genetic family tree.

Short stature, high‐arched palate, widely spaced teeth with malocclusion, and bridged palmar crease of the left hand can be observed in the patient (Figure 2).

### 2.2 Adjuvant examination results

The patient's chromosomes were 46, XX, and the copy‐number variants (CNVs) were also normal. Her fasting insulin (FI) level was 1.1 uIU/mL (reference value: 3.0–25.0 uIU/mL), insulin‐like growth factor‐1 (IGF‐1) level was 51.8 ng/mL (reference value: 83.0–691.0 uIU/mL), and IGF‐1 bp3 level was 2.72 µg/mL (reference value: 2.39–13.8 µg/mL); all these levels were decreased compared with the respective references. The growth hormone provocation test revealed a peak value of 8.1 ng/mL (reference value: >15 ng/mL). The Wechsler intelligence scale measured her intelligence quotient (IQ) at the age of 9 as 43, meaning she has hypophrenia. The Child Behavior Checklist (CBCL) revealed that the proband had severe maladjusted behavior, including obvious social withdrawal, schizoid personality disorder, inattention, hyperactivity, destructive behavior, and maltreatment. Her skeletal age was 9 years and 11 months old, which lagged her actual age. The complete blood count, routine urine‐check, routine excrement examination, adrenocorticotropic hormone, plasma cortisol level, blood lipid level, HbA1c, the thyroid function test, electroencephalogram (EEG), ultrasonic testing of the abdomen and urinary systems, and brain CT were all normal. The anterior‐posterior and lateral films of the whole spine were normal. Her parents were both healthy. She has a 24‐year‐old half‐brother with the same father who was diagnosed with epilepsy. Our patient has never suffered from epileptic seizures. The proband's parents denied any history of inherited metabolic disease.

## 3 METHODS

### 3.1 Ethical compliance

Our study was approved by the Ethics Committee of West China Second Hospital of Sichuan University. We obtained written informed consent from the patient's parents prior to conducting the WES. Informed consents were obtained from patient's parents for inclusion of her clinical and imaging details in the manuscript for the purpose of publication.

### 3.2 DNA extraction

The umbilical cord blood or fetal tissue genomic DNA was extracted using the Blood Genome Column Medium Extraction Kit (Kangweishiji, China) according to the manufactural instructions. The extracted DNA samples were subjected to quality controlling using Qubit 2.0 fluorimeter and electrophoresis with 0.8% of agarose gel for further protocol.

### 3.3 Whole‐exome library construction

Protein‐coding exome enrichment was performed using the xGen Exome Research Panel v1.0 (IDT), consisting of 429,826 individually synthesized and quality‐controlled probes, targeting 39 Mb protein‐coding regions (19,396 genes) of the human genome and covering 51 Mb of end‐to‐end tiled probe space.

### 3.4 Sequencing

High‐throughput sequencing was performed using the Illumina NovaSeq 6000 series sequencer (PE150). Nothing less than 99% of the target sequence was sequenced.

Bioinformatics analysis

1. Quality control. Raw data were processed using fastp for adapters removing and low‐quality reads filtering.
2. Variants calling. The paired‐end reads were performed using the Burrows–Wheeler Aligner (BWA) to the Ensembl GRCh37/hg19 reference genome. Base quality score recalibration, together with single‐nucleotide variants (SNPs), short indel calling, and CNVs were conducted using GATK. High quality and reliable variants were obtained according to the sequence depth and variant quality, SNPs, Indels, and CNVs.
3. Variant annotation and pathogenicity prediction. The online system independently developed by Chigene (www.chige​ne.org) was used to annotate the database‐based minor allele frequencies (MAFs) and ACMG (American College of Medical Genetics) practice guideline‐based pathogenicity for every gene variant. The system also provided serial software packages for conservative analysis and protein product structure prediction. The databases for the annotation of MAFs include 1,000 genomes, dbSNP, ESP, ExAC, and Chigene in‐house MAFs database; Provean, Sift, Polypen2_hdiv, Polypen2_hvar, MutationTaster, M‐Cap, and Revel software packages were used to predict protein product structure variation. The prioritized pathogenicity annotation of ACMG guidelines, the OMIM, HGMD, and ClinVar databases were used as conferences for pathogenicity of every variant. To predict the functional changes associated with splice variants, the MaxEntScan, dbscSNV, and GTAG software packages were used instead of the product structure prediction software.

## 4 MOLECULAR RESULTS

**FIGURE 3:**
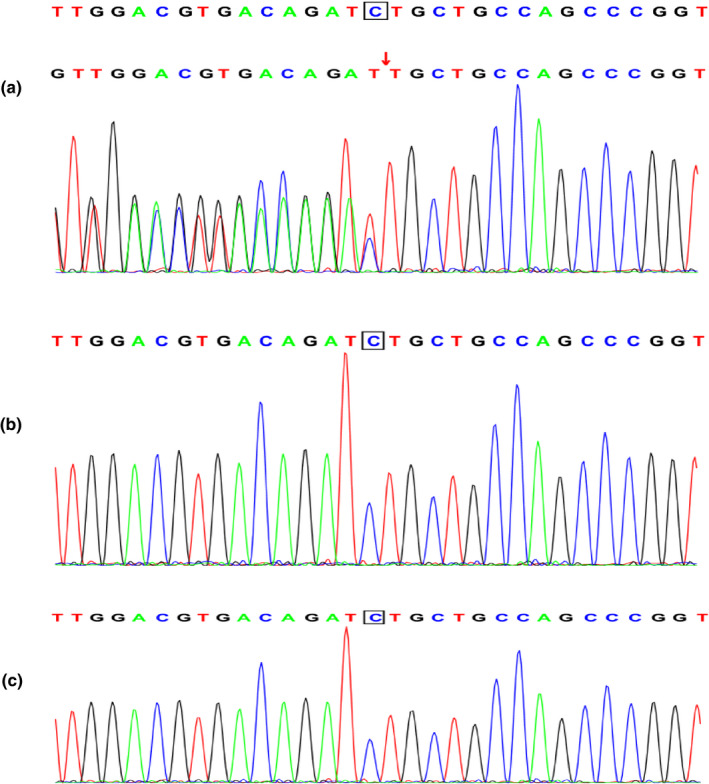
Results of the *SON* analysis in the patient and her parents. (a) A heterozygous variant c.5297del in *SON* was detected in the child. (b) No variant in *SON* was detected in the father of the child. (c) No variant in *SON* was detected in the mother of the child.

A novel variant, c.5297del, that causes a p.Ser1766Leufs*7 frameshift was detected in *SON* in the proband. No variant was detected in the patient's parents and their genotype was normal (Figure 3). Sanger sequencing was used to verify the de novo variant in the proband and her parents. According to the 2015 ACMG guidelines, the variant meets the criteria to be identified as a pathogenic variant: PVS1+PS2+PM2.

## 5 DISCUSSION

The clinical features of our patient include: short stature, intellectual disability, developmental delay, high‐arched palate, widely spaced teeth with malocclusion. Notably, unlike other reported cases, our patient had a bridged palmar crease on her left hand and presented with low growth hormone levels when she was 7 years and 3 months old.

All reported (33) cases of ZTTK syndrome had intellectual disability, developmental delay, and facial dysmorphism. Brain malformations (ventricular enlargement, thin corpus callosum, abnormal cerebral cortical gyration, arachnoid cyst, cerebellar dysplasia, and/or white matter abnormality) were observed in 85.9% of the subjects. Neurological features such as hyper‐or hypotonia, epilepsy or other EEG abnormalities, and autism spectrum disorder occurred in 62.0% of the subjects. Musculoskeletal abnormalities (joint laxity, scoliosis, or kyphosis, hemivertebrae, cubitus valgus, contractures, small hands and feet, and/or abnormal ribs) were identified in 77.3% of the subjects. Approximately 69.6% of the cases had eye and/or vision abnormalities, of which strabismus was the most common. Others include hyperopia, cortical visual impairment, and optic atrophy. Short stature occurred in 51.8% of the cases. Other manifestations include heart defects (ventricular/atrial septal defect and patent ductus arterious), urogenital malformations (single kidney, horseshoe kidney, and kidney dysplasia), intestinal atresia, high position or cleft palate, craniosynostosis. Several subjects have feeding difficulties early after birth, some even require a gastrostomy feeding tube. Immunoglobulin deficiency and abnormal coagulation also occur in several patients (Kim, Baddoo, et al., 2016; Kim, Shinde, et al. 2016; Takenouchi et al., 2016; Tokita et al., 2016; Yang et al., 2019; Zhu et al., 2015). We have summarized the clinical features (common features vs. unique features) of patient in our report and cases having been reported to date in Table 1. So far, 26 causative *SON* variants have been identified in ZTTK syndrome, of which 25 are in exon 3. In all 33 reported subjects, the c.5753_5756del and c.3852_3856del variants were detected in eight and two subjects, respectively. The rest of the 24 causative variants have only been identified once. There are two subjects who each carrying two variants in their *SON*. There is one subject carrying a small deletion of copy‐number variant, including *SON* and five other genes.

**TABLE 1:** Clinical features of the ZTTK syndrome reported to date.

Clinical features	Zhu et al. (2015)	Kim, Baddoo, et al., (2016); Kim, Shinde, et al. (2016)	Takenouchi et al. (2016)	Tokita et al. (2016)	Yang et al. (2019)	Quintana et al. (2020)	Tan et al. (2020)	Present study (2020)
Developmental delay/Intellectual disability	+	+	+	+	+	+	+	+
Facial dysmorphism	+	+	+	+	+	+	+	
Short stature		+	+	+	+	+	+	+
Brain malformation	+	+		+	+	+	+	
Ventricular enlargement		+					+	
Corpus callosum abnormality		+		+	+		+	
Cortex malformation		+						
White matter abnormalities	+	+			+	+		
Cerebellar abnormalities		+						
Seizures	+	+	+	+			+	
Musculoskeletal abnormalities		+		+	+			
Eye and/or vision abnormality		+		+		+		
Gastrointestinal malformation	+	+						
Urogenital malformation		+		+				
Heart defect	+	+	+	+		+		
Bridged palmar crease								+
Growth hormone deficiency								+

The *SON* gene consists of 12 exons, with the size of exon 3 accounting for 82% of the entire‐coding region (Khan et al., 1994). This gene plays an essential role in both constitutive and alternative splicing, especially in splicing short introns with suboptimal weak splice sites (Ahn et al., 2011; Hickey, Kim, & Ahn, 2014; Lu et al., 2013). The Residual Variation Intolerance Score of *SON* is 1.88, meaning that it belongs to the 2% of the most intolerant human protein‐coding genes (Petrovski, Wang, Heinzen, Allen, & Gold‐stein, 2013). When *SON* was knocked down in HeLa and human embryonic stem cells, a group of genes essential to neuronal cell migration, embryonic survival, metabolism, and mitochondrial function, including *TUBG1*, *FLNA*, *PNKP*, *WDR62*, *PSMD3*, *HDAC6*, *PCK2*, *PFKL*, *IDH2*, *ACY1*, and *ADA* showed significantly decreased expression (Ahn et al., 2011; Kim, Baddoo, et al., 2016; Kim, Shinde, et al. 2016; Lu et al., 2013). These genes play essential roles in many aspects of human growth and development (Alejandro et al., 2018; Li, Xie, Xiao, & Wang, 2020; Mona, Masoumeh, Saeed, Mehran, & Mohammad, 2019; Poirier et al., 2013). Thus, variants in *SON* result in severe and extensive multi‐systematic detriments.

Recently, Ueda et al. demonstrated that *SON* knockdown in mouse neural progenitors resulted in defective migration during corticogenesis and reduced the spine density of mature cortical neurons. The induction of human wild‐type SON expression rescued these neural abnormalities, confirming that the abnormalities were caused by SON insufficiency. In addition, this data supports the idea that the neural abnormalities in ZTTK syndrome are caused by SON haploinsufficiency rather than functional or dysfunctional proteins resulting from different types of mutations (Ueda et al., 2020).

In conclusion, we report an additional case of ZTTK syndrome and reveal a novel disease‐causing variant in *SON*. Also, we emphasize the importance of WES for early diagnosis of ZTTK syndrome, which can speed up the diagnostic procedure, sparing patients from unnecessary investigations and ineffective, or even harmful treatments. Our report expands the mutant spectrum of the *SON* gene and refines the genotype‐phenotype map of ZTTK syndrome.

## CONFLICT OF INTEREST

We declare that we have no competing interests.

## ETHICAL APPROVAL STATEMENT

Our study was approved by the Ethics Committee of West China Second Hospital of Sichuan University. With written informed consent from parents of the patient, we conducted the genetic tests. Informed consent was obtained from the parents of our patient for inclusion of her clinical and imaging details in the manuscript for the purpose of publication.

## Supporting information

Table S1

## Data Availability

Data sets used in this study are available from the corresponding author upon reasonable request.
