# Supplementary material for: A de novo heterozygous variant in the SON gene is associated with Zhu‐Tokita‐Takenouchi‐Kim syndrome
Source: Mol Genet Genomic Med. 2020 Sep 14;8(11):e1496. doi: 10.1002/mgg3.1496 (PMC7667370; doi:10.1002/mgg3.1496)
Supplement: Supplementary file 1 — Table S1 [file MGG3-8-e1496-s001.docx]

**Table S1. HGVS-compliant Descriptions of variants in Our Report**

variants in our report HGVS-compliant variants descriptions

c.268del NM_138927.2:c.268del / NC_000021.9:g.33549499del

c .286C>T NM_138927.2:c.286C>T / NC_000021.9:g.33549517C>T

c.394C>T NM_138927.2:c.394C>T / NC_000021.9:g.33549625C>T

c.1444del NM_138927.2:c.1444del / NC_000021.9:g.33550675del

c.1881_1882del NM_138927.2:c.1881_1882del / NC_000021.9:g.33551112_33551113del

c.2365del NM_138927.2:c.2365del / NC_000021.9:g.33551596del

c.3073dup NM_138927.2:c.3073dup / NC_000021.9:g.33552304dup

c.3334C>T NM_138927.2:c.3334C>T / NC_000021.9:g.33552565C>T

c.3556C>T NM_138927.2:c.3556C>T / NC_000021.9:g.33552787C>T

c.3597_3598dup NM_138927.2:c.3597_3598dup / NC_000021.9:g.33552828_33552829dup

c.3852_3856del NM_138927.2:c.3852_3856del / NC_000021.9:g.33553083_33553087del

c.4055del NM_138927.2:c.4055del / NC_000021.9:g.33553286del

c.4151_4174del NM_138927.2:c.4151_4174del / NC_000021.9:g.33553382_33553405del

c.4358_4359del NM_138927.2:c.4358_4359del / NC_000021.9:g.33553589_33553590del

c.4549dup NM_138927.2:c.4549dup / NC_000021.9:g.33553780dup

c.4640del NM_138927.2:c.4640del / NC_000021.9:g.33553871del

c.4909A>T NM_138927.2:c.4909A>T / NC_000021.9:g.33554140A>T

c.4999_5013del NM_138927.2:c.4999_5013del / NC_000021.9:g.33554230_33554244del

c.5031_5032insAA NM_138927.2:c.5031_5032insAA / NC_000021.9:g.33554262_33554263insAA

c.5230del NM_138927.2:c.5230del / NC_000021.9:g.33554461del

c.5297del NM_138927.2:c.5297del / NC_000021.9:g.33554528del

c.5528C>A NM_138927.2:c.5528C>A / NC_000021.9:g.33554759C>A

c.5549_5550del NM_138927.2:c.5549_5550del / NC_000021.9:g.33554780_33554781del

c.5753_5756del NM_138927.2:c.5753_5756del / NC_000021.9:g.33554984_33554987del

c.6002_6003insCC NM_138927.2:c.6002_6003insCC / NC_000021.9:g.33555233_33555234insCC

c.6087del NM_138927.2:c.6087del / NC_000021.9:g.33555318del

c.6233del NM_138927.2:c.6233del /NC_000021.9:g.33557228del

Red words are *de novo* variants revealed in our report.
